# Commentary on: “A common origin for immunity and digestion”

**DOI:** 10.3389/fmicb.2015.00531

**Published:** 2015-05-27

**Authors:** Gustav van Niekerk, Anna-Mart Engelbrecht

**Affiliations:** Department of Physiological Sciences, Stellenbosch UniversityStellenbosch, South Africa

**Keywords:** adaptive immune system, evolutionary constraint, intestinal biota, immunological novelty

We read with great interest the recent hypothesis forwarded by Broderick (2015). The underlying question addressed in her thesis is central to the field of evolutionary immunology: What drives immunological innovation? This question finds its most intriguing manifestation in what has been called “arguably the most exciting finding of the past decade in immunology” (Flajnik, 2014): the discovery of a second, convergent evolved, adaptive immune system (AIS) in jawless vertebrates (hagfish and lampreys). This discovery raises the question, why did vertebrates, (representing about 1% of all animals that ever lived) evolve an AIS twice, whereas invertebrates failed to do so? Clearly, understanding the forces that drive immunological innovation will undoubtedly have a formative impact on our understanding of one of the most perplexing immunological dimorphisms in nature.

Broderick argues that both immune and digestive systems might have shared a common origin and goal: “the quest for more efficient energy acquisition” (Broderick, 2015). The author draws on two lines of arguments to support her claim. Firstly, she points out that various “enzymes involved in immune responses also [perform] roles in digestion.” Secondly, she notes that the gut represented an early innovation in metazoans, from which, as is hypothesized, immune functionality developed. Indeed, the author notes that this connection between immunity and digestion might be ancient since, “for single-celled organisms like amoebae, the process of infection and food acquisition are indistinguishable in the initial stages, with the two being separable only by outcome.” Though, this may be true for single cell organism (and very primitive animals such as sea sponges), we are skeptical of the extent to which the sharing of similar components (i.e., digestive enzymes) between systems justifies an interpretation of common ontology between digestion and immune system in higher animals.

Both immune and digestive systems make use of proteolytic enzymes, but often for vastly different reasons. Beyond playing a role in direct pathogen killing, proteolytic enzymes also perform a range of other functions within an immunological context. As an example, neutrophils failing to detect pathogens after being activated proceed to liquefy surrounding tissue by the release of proteases in order to gain access to pathogens (Nathan, 2006). Furthermore, proteolytic enzymes also activate pro-antimicrobial peptides [e.g., proteinase 3 processing pro-AMP into mature LL-37 (Sorensen et al., 2001)] and orchestrate signaling context by processing signaling molecules [e.g., IL-1β, IL-6, and IL-18 (Meyer-Hoffert and Wiedow, 2011)].

Considering these differential requirements between immune and digestive systems, we suspect that digestive enzymes (and their predecessors) evolved in parallel (i.e., concurrent in both the digestive and the immune system), probably “swapping” proteases a number of times (Figure 1). Thus, instead of ascribing the recruitment of similar proteins in different systems to shared ontology, we argue that both systems co-opted the application of similar digestive enzymes as these two systems shared a common need to break down (i.e., digest) various molecules. Sequential innovation might give rise to an enzyme that can also realize a function within another system. Consequently, once such an innovation arises, the same protein can opportunistically be incorporated into other systems.

**Figure 1:**
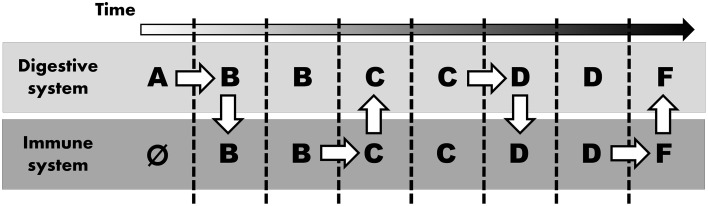
**Parallel evolution mimicking common ontology**. Alphabetic letters represent stepwise modification to digestive enzyme (starting at A, arbitrarily assigned to the digestive system and immune system lacking any digestive enzyme initially, as indicated by Ø). White arrows indicate “evolutionary innovation”: mutation (horizontal arrow) or novel application in any other system (vertical arrow).

Furthermore, we wish to comment on the role of pathogen stress as the impetus for immunological innovation. We believe that a large body of evidence indicates that pathogen stress would suffice as an evolutionary drive toward immunological innovation and that other explanations (such as a drive toward more economical resource acquisition –as argued by Broderick) are complementary at best, but not necessary. By all accounts, pathogens have left their mark on host genomes [including humans (Barreiro and Quintana-Murci, 2010)], with genomic sequencing of various animals repeatedly demonstrating selective pressure operating on immune genes. Furthermore, the AIS arose in marine animals (before the transition to land): Sea water contains a much larger concentration of pathogens [e.g., bacteria at a concentration of 105–107/ml and sediments 108–1010/g (Austin, 1988; Otero-Gonzalez et al., 2010)] than the terrestrial environment. Taken together, we reason that all multicellular life experiences a constant drive for immunological innovation by virtue of continual pathogen stress.

Arguments for evolutionary novelty arising from serendipitous events such as the two rounds of whole genome duplication in vertebrates (Flajnik and Kasahara, 2009), and the incorporation of either bacterial (Flajnik and Kasahara, 2009) or viral (Zhang et al., 2014) transposable elements, giving rise to *Rag* genes, have been proposed as the drivers of AIS. However, the discovery of *Rag* gene candidates in invertebrates (Fugmann et al., 2006; Holland et al., 2008), as well as the fact that the VLR-based AIS of jawless vertebrates function without the *Rag* gene (Boehm et al., 2012) strongly suggest that *Rag* genes are neither necessary nor sufficient for the development of AIS. As for genome duplication, these events do not explain the emergence of evolutionary novelty, but “merely enhances the diversification potential of a lineage” (Van de Peer et al., 2009) and thus do not present an explanation for why “the diversification potential” should manifest in the emergence of AIS. In addition, it has become apparent that evolutionary novelty are more often than not generated by the “modification of pre-existing genetic regulatory circuits” (Shubin et al., 2009) which can provide for ample structural variety (Carroll, 2008). We also believe that this process provides a more parsimonious explanation for the shared commonality observed between the digestive and immune systems: reinventing novel application of pre-existing genes is more likely than evolving novel genes with similar functionality.

Broderick also refers to McFall-Ngai's (2007) theory which points out that the need to cultivate symbiotic relationships with intestinal biota provided the evolutionary drive toward the development of an AIS. However, this raises the question: if the AIS evolved to manage prokaryotic symbiotes, why did invertebrates not similarly develop an AIS? After all, invertebrates also interact with various symbiotes (Dillon and Dillon, 2004; Ruby, 2008; Ratzka et al., 2012; Degnan, 2015). Instead, we suspect that the AIS arose due to pathogen stress and only after its establishment, opportunistically acquired the function of managing intestinal biota later on. In other words, managing intestinal biota with the aid of AIS is *a result of* rather than *a cause of* AIS.

However, an insistence on pathogen stress as a driver of immunological novelty invokes the same question: if pathogen stress is what is driving immunological innovation, why have invertebrates not similarly developed an AIS? We suspect that, instead of focusing on the evolutionary pressure that “drives” immunological innovations, it might be productive to explore the evolutionary constraints that hamper the deployment of immunological novelties. With regards to AIS, we argue that the advent of adipocytes might have provided the evolutionary release by expanding metabolic scope and thus allowed the implementation of a metabolically costly AIS in vertebrates (van Niekerk and Engelbrecht, 2015). Since “executing the immunological mandate requires more than just an immune system” (van Niekerk and Engelbrecht, 2015), it is likely that other physiological parameters of invertebrates might prevent certain immunological innovations. Furthermore, we believe that understanding why certain immunological innovations remain an “un-evolvable trait” may expand our understanding of how immune and none-immune systems “functionally synapse” on each other.

## Conflict of interest statement

The authors declare that the research was conducted in the absence of any commercial or financial relationships that could be construed as a potential conflict of interest.

## References

[B1] Austin B. (1988). Marine Microbiology. Cambridge: CUP Archive.

[B2] Barreiro L. B. Quintana-Murci L. (2010). From evolutionary genetics to human immunology: how selection shapes host defence genes. Nat. Rev. Genet. 11, 17–30. 10.1038/nrg2698 19953080

[B3] Boehm T. McCurley N. Sutoh Y. Schorpp M. Kasahara M. Cooper M. D. (2012). VLR-based adaptive immunity. Annu. Rev. Immunol. 30, 203–220. 10.1146/annurev-immunol-020711-075038 22224775 PMC3526378

[B4] Broderick N. A. (2015). A common origin for immunity and digestion. Front. Immunol. 6:72. 10.3389/fimmu.2015.00072 25745424 PMC4333870

[B5] Carroll S. B. (2008). Evo-devo and an expanding evolutionary synthesis: a genetic theory of morphological evolution. Cell 134, 25–36. 10.1016/j.cell.2008.06.030 18614008

[B6] Degnan S. M. (2015). The surprisingly complex immune gene repertoire of a simple sponge, exemplified by the NLR genes: a capacity for specificity? Dev. Comp. Immunol. 48, 269–274. 10.1016/j.dci.2014.07.012 25058852

[B7] Dillon R. Dillon V. (2004). The gut bacteria of insects: nonpathogenic interactions. Annu. Rev. Entomol. 49, 71–92. 10.1146/annurev.ento.49.061802.123416 14651457

[B8] Flajnik M. F. (2014). Re-evaluation of the immunological Big Bang. Curr. Biol. 24, R1060–R1065. 10.1016/j.cub.2014.09.070 25517375 PMC4354883

[B9] Flajnik M. F. Kasahara M. (2009). Origin and evolution of the adaptive immune system: genetic events and selective pressures. Nat. Rev. Genet. 11, 47–59. 10.1038/nrg2703 19997068 PMC3805090

[B10] Fugmann S. D. Messier C. Novack L. A. Cameron R. A. Rast J. P. (2006). An ancient evolutionary origin of the Rag1/2 gene locus. Proc. Natl. Acad. Sci. U.S.A. 103, 3728–3733. 10.1073/pnas.0509720103 16505374 PMC1450146

[B11] Holland L. Z. Albalat R. Azumi K. Benito-Gutierrez E. Blow M. J. Bronner-Fraser M.. (2008). The amphioxus genome illuminates vertebrate origins and cephalochordate biology. Genome Res. 18, 1100–1111. 10.1101/gr.073676.107 18562680 PMC2493399

[B13] McFall-Ngai M. (2007). Adaptive immunity: care for the community. Nature 445, 153–153. 10.1038/445153a 17215830

[B14] Meyer-Hoffert U. Wiedow O. (2011). Neutrophil serine proteases: mediators of innate immune responses. Curr. Opin. Hematol. 18, 19–24. 10.1097/MOH.0b013e32834115d1 21042214

[B15] Nathan C. (2006). Neutrophils and immunity: challenges and opportunities. Nat. Rev. Immunol. 6, 173–182. 10.1038/nri1785 16498448

[B16] Otero-Gonzalez A. J. Magalhaes B. S. Garcia-Villarino M. Lopez-Abarrategui C. Sousa D. A. Dias S. C.. (2010). Antimicrobial peptides from marine invertebrates as a new frontier for microbial infection control. FASEB J. 24, 1320–1334. 10.1096/fj.09-143388 20065108

[B17] Ratzka C. Gross R. Feldhaar H. (2012). Endosymbiont tolerance and control within insect hosts. Insects 3, 553–572. 10.3390/insects3020553 26466544 PMC4553611

[B18] Ruby E. G. (2008). Symbiotic conversations are revealed under genetic interrogation. Nat. Rev. Microbiol. 6, 752–762. 10.1038/nrmicro1958 18794913 PMC3579588

[B19] Shubin N. Tabin C. Carroll S. (2009). Deep homology and the origins of evolutionary novelty. Nature 457, 818–823. 10.1038/nature07891 19212399

[B20] Sorensen O. E. Follin P. Johnsen A. H. Calafat J. Tjabringa G. S. Hiemstra P. S.. (2001). Human cathelicidin, hCAP-18, is processed to the antimicrobial peptide LL-37 by extracellular cleavage with proteinase 3. Blood 97, 3951–3959. 10.1182/blood.V97.12.3951 11389039

[B21] Van de Peer Y. Maere S. Meyer A. (2009). The evolutionary significance of ancient genome duplications. Nat. Rev. Genet. 10, 725–732. 10.1038/nrg2600 19652647

[B22] van Niekerk G. Engelbrecht A. (2015). On the evolutionary origin of the adaptive immune system-the adipocyte hypothesis. Immunol. Lett. 10.1016/j.imlet.2015.02.002 25698354

[B23] Zhang Y. Xu K. Deng A. Fu X. Xu A. Liu X. (2014). An amphioxus RAG1-like DNA fragment encodes a functional central domain of vertebrate core RAG1. Proc. Natl. Acad. Sci. U.S.A. 111, 397–402. 10.1073/pnas.1318843111 24368847 PMC3890805

